# Beck’s Lectures on Materia Medica and Therapeutics

**Published:** 1852-06

**Authors:** 


### EDITORIAL.

*Lectures on Materia Medica and Therapeutics, delivered in the College of Physicians and Surgeons of the University of the State of New York.* By JOHN B. BECK, M. D., late Professor of Materia Medica and Medical Jurisprudence. Prepared for the press by his friend C. R. GILMAN, M.D., Professor of Obstetrics, etc., in the College of Physicians and Surgeons, N. Y. pp. 582, octavo: New York: Samuel S. & William Wood, 261 Pearl street; 1851. From the publishers, through A. H. & C. Burley.

THE work with the above title, like all those from the justly celebrated publishing house of the Messrs. Wood, is, as regards its mechanical execution, creditable to the publishers. Of its contents, however, we cannot as unhesitatingly express a favorable opinion.

As indicated by the title, it is simply a printed copy of Dr. Beck's lectures on Materia Medica and Therapeutics prepared previous to the discovery and adoption of many of the more modern improvements in this department of medicine, and therefore contains the views and opinions entertained by the profession at that time; but in our judgment falls far short of other works already in the hands of physicians—such as Pereira's, for instance—in giving an exposition of the present state of knowledge upon this subject.

In his preface, the editor, Dr. Gilman, remarks as follows:

"On examining the manuscripts I found proof of a fact of which I was before cognisant, viz.: that on many of what are called the 'New Remedies,' Dr. Beck did not lecture. He was in truth, as these lectures in almost every page will prove, not a runner after new things; his study was much more into the indications of treatment, the circumstances modifying the operation of medicines, and those kindred topics which I should call the philosophy of Materia Medica, than into the character and claims of new and fashionable therapeutic agents. This explains the fact that many 'new things' found no place in his lectures. I had no disposition to alter them. In this respect I shared his opinions, and concurred heartily in his plan of teaching."

We can readily conceive why views such as the above were entertained by an old practitioner and teacher like Dr. Beck, but cannot account for the want of confidence in modern improvements thus expressed by Dr. Gilman, who, in the prime of life, and in the great commercial emporium of our country, must have seen and learned from his own experience and observation the almost incalculable advantages which may be derived from medicines but recently brought into use, such as cod liver oil, chloroform, the vegetable alkalies, acids, &c.

Our cotemporary, Dr. Flint, of the Buffalo *Medical and Surgical Journal*, gave to its readers not long since a truthful and graphic description of "monumental physicians." In our opinion, the work before us is justly entitled to a place in the libraries of all practitioners of this class, for it is in fact a "monumental" book, which will, to say the least, serve to transmit to posterity the memory of Dr. Beck, and represent the state of therapeutical knowledge in his time.

Believing as we do that most, if not all, judicious practitioners of the present day, with ourselves, regard opium in large doses as one of the most efficient remedial agents in all inflammatory affections, we will leave our readers to judge, after reading the following quotation, if or not those of the author are antiquated doctrines:

"Now the first remark that I would make is, that opium does not exercise any control at all over inflammatory action. It quiets pain and produces insensibility. But this is all that it can do, and this only temporarily. Besides, after the effect of the opium passes off, the inflammation, so far from being relieved, is generally made worse, and the reason is obvious from the very effects of opium. Instead of evacuating the inflamed vessels, opium checks the secretions, and in this way still further engorges them. As a remedy, then, *per se*, in inflammation, it is not merely useless, but injurious. In this way, therefore, it is never to be used. As an adjuvant, however, to other remedies, it may be made exceedingly useful, and should never be neglected. Now, the objects for which we use it as an adjuvant, are: 1. To allay the reaction which generally succeeds the loss of blood in inflammation. 2. To allay pain and irritation consequent upon inflammation. To accomplish these objects there are two general modes in which we use it. By combining venesection and the use of calomel and tartar emetic with opium. Now the rationale of this combination is the following, and is perfectly philosophical. The venesection reduces the inflammatory action, and brings the system into a condition in which opium produces simply its sedative and soothing operation, and in this way it acts most kindly on the disease. Then, again, the calomel and tartar-emetic co-operate most powerfully with the venesection in subduing inflammatory action, keeping down the pulse, and promoting all the secretions. By these various means the system is kept in a state in which the opium may be safely used, and in this way you get from it the only effect which you wish to obtain, i. e. its soothing effects, while you rob it of all its exciting and disturbing qualities.

"Another mode of using opium in inflammation is so to subdue the inflammatory excitement, as that nothing need be apprehended from the stimulant or astringent operation of the opium, but that only the sedative effects shall follow. This is done by bleeding at first to deliquium, and then giving a large dose of opium. If the symptoms return, the same may be repeated, and to remove the remaining traces of inflammation, calomel and ipecac, with the opium in smaller doses may be resorted to. This is the practice recommended by Dr. Armstrong. (Lect. 394.) Now, the principle upon which he used opium is simply this. If you merely bleed a patient laboring under inflammation, however copiously, without following it up by any other remedy, you will find that in a short time he recovers from the effects of it, reaction takes place, and the inflammatory excitement is renewed, although perhaps in a milder degree. To subdue this, further depletion will be found necessary. If, on the contrary, after free depletion you give a large dose of opium, it quiets the irritability of the system, vascular and nervous, upon which the renewal of the inflammation depends, and in this way the subsequent excitement is rendered so moderate as to be manageable by the other remedies just mentioned. This is a kind of treatment which you will find very useful in cases where it is important to husband the strength of your patient as it regards the loss of blood. Most patients, however delicate, will bear one good bleeding, while repeated bleedings may exhaust them.

"Now, if you reflect upon these two modes of using opium, you must see, I think, that opium has no direct agency in controlling the inflammation at all. This is done by the venesection, tartar emetic, ipecacuanha, and calomel; and the only effect of the opium is to soothe and prevent reaction. In both these respects, however, it is invaluable, and if used with discretion and judgment, may aid wonderfully in conducting the case to a favorable issue.

"This, then, is the principle upon which I think opium ought to be used in inflammation—not to arrest inflammation itself, but to control some of the effects of it. If this be correct, it must be obvious that it ought never to be used in the early stages of inflammatory complaints. In consequence of not attending to this, it is to be feared that many a case of inflammation has terminated fatally; commencing insidiously—perhaps mistaken for a spasm—opium has been given, the patient has apparently been relieved, until in a short time inflammation has developed itself in such a way as to be uncontrollable. In all cases, therefore, where you are in doubt about a case being inflammation or spasm, be cautious in the use of opium. It cloaks up the disease in such a way that you may not be able to distinguish it before it is too late.

"Although the principle upon which we use opium is perhaps the same in all cases of inflammation, yet it is greatly modified in its application by various circumstances, and they must be specially attended to. The principal of these is the locality of the inflammation, not merely as regards the tissue which is the seat of it, but the particular part of the tissue." H.

